# Preparation and Characterization of Rice Bran Protein Hydrolysates Enhanced via Alcalase and *Lactiplantibacillus plantarum* 13110 Co-Treatment: Antioxidant Properties and Ameliorative Effects on Ulcerative Colitis in Mice

**DOI:** 10.3390/nu18081278

**Published:** 2026-04-17

**Authors:** Guanlong Li, Xiaolan Liu, Peng Li, Quanxin Wang, Changyuan Wang, Xiqun Zheng

**Affiliations:** 1 Heilongjiang Provincial Key Laboratory of Corn Deep Processing Theory and Technology, College of Food and Bioengineering, Qiqihar University, Qiqihar 161006, China; 03580@qqhru.edu.cn (G.L.); 19056140312@163.com (P.L.); wangquanxin979@163.com (Q.W.); 2 Engineering Research Center of Plant Food Processing Technology, Ministry of Education, Qiqihar University, Qiqihar 161006, China; 3 College of Food Science, Heilongjiang Bayi Agricultural University, Daqing 163319, China; 18814720081@163.com

**Keywords:** rice bran protein hydrolysates, ulcerative colitis, *Lactiplantibacillus plantarum* 13110, intestinal barrier function

## Abstract

Background: Ulcerative colitis, a chronic inflammatory disorder of the intestine, represents a major health concern worldwide. This study aimed to explore the in vivo efficacy of rice bran protein hydrolysates in mitigating UC. Methods: Rice bran protein hydrolysates with enhanced antioxidant activity were prepared via co-treatment with Alcalase and *Lactiplantibacillus plantarum* 13110. Results: Compared with hydrolysates obtained using Alcalase in isolation (RHP), the co-treated rice bran (CRB) protein hydrolysates exhibited significantly higher antioxidant capacity. Structural characterization revealed marked alterations in molecular weight distribution, amino acid composition, and RHP spectral features, based on Fourier transform infrared spectroscopy, during fermentation with *L. plantarum* 13110. The 500 mg/kg·bw CRB intervention effectively attenuated oxidative stress and inflammatory responses in dextran sulfate sodium (DSS)-induced colitic mice, as evidenced by significantly reduced colonic levels (*p* < 0.05) of pro-inflammatory mediators (TNF-α, IL-1β, IL-6, and LPS), decreased serum concentrations of fatty acid-binding protein 2 (FABP2), diamine oxidase (DAO), and D-lactic acid (D-LA), and increased colonic IL-10 content (*p* < 0.05). These changes were associated with ulcerative colitis amelioration and improved intestinal barrier function. Conclusions: Thus, CRB exhibits promising prophylactic effects against ulcerative colitis, suggesting its potential for therapeutic application.

## 1. Introduction

Ulcerative colitis, a chronic and recurrent inflammatory bowel disease, poses a growing global health burden due to its increasing incidence and severe detriment to patient well-being [1]. The pathological features of ulcerative colitis include persistent inflammation of the colonic mucosa, damage to the intestinal barrier, and immune system dysfunction [2]. Currently, clinical treatment primarily relies on aminosalicylic acid preparations, glucocorticoids, and immunosuppressants. However, these medications often come with significant limitations, including side effects, high recurrence rates, and substantial costs [3]. Consequently, identifying safe, effective, and widely available natural products or foods that prevent or alleviate colon inflammation has become one of the current research hotspots. Given that oxidative stress plays a fundamental role in the development and progression of ulcerative colitis, strategies aimed at mitigating oxidative damage have attracted considerable research interest [4]. During intestinal inflammation, activated immune cells generate excessive reactive oxygen species (ROS), while an imbalance in the endogenous antioxidant defense system leads to redox homeostasis disruption [5]. Alleviating intestinal oxidative stress and restoring gut health are crucial for maintaining intestinal barrier integrity. Exogenous antioxidant intervention is among the effective strategies for alleviating colitis. Although oxidative stress is recognized as a central driver in the pathogenesis of ulcerative colitis (UC), the clinical evidence supporting antioxidant interventions remains limited and methodologically fragile. A meta-analysis reported that antioxidant supplementation significantly improved oxidative stress markers and modestly reduced disease activity scores but did not improve quality of life [6]. These findings suggest that current antioxidant strategies may only play an adjunctive—not primary—therapeutic role in UC management. Given these clinical uncertainties, rigorous preclinical studies remain essential to identify novel antioxidant candidates with higher efficacy and to elucidate their molecular mechanisms before advancing to well-designed, adequately powered clinical trials.

Rice bran, as the primary byproduct of rice processing, is rich in high-quality protein, which is mainly composed of crushed rice, the ectoderm, and the rice cortex [7]. The protein content in rice bran is about 15–17% and mainly comprises albumin (12.5–43%), globulin (13–36%), prolamin (1–5%), and glutelin (22–45%) [8]. Rice bran protein possesses an amino acid composition superior to that of other cereal proteins and closely approximates the reference pattern established by the FAO/WHO [9]. Therefore, rice bran protein has significant advantages for researchers investigating novel plant protein resources. Studies have found that the peptides derived from rice bran proteins possess some therapeutic properties, such as antioxidant [10], antihypertensive [11], immune regulation [12], and anti-cancer activity [13], and can be obtained via protease hydrolysis or microbial fermentation of rice bran protein.

Specifically, accumulating evidence indicates that rice bran protein hydrolysates exhibit robust antioxidant and anti-inflammatory activities. He et al. [14] demonstrated that their anti-inflammatory mechanism involves modulating the MAPK signaling pathways. In line with this, Boonloh et al. [15] showed that hydrolysates from rice bran protein significantly suppressed pro-inflammatory mediators in mice fed a high-carbohydrate diet, underscoring their anti-inflammatory potential. Collectively, these results imply that rice bran protein hydrolysates may counteract free radical-induced oxidative injury and ameliorate oxidative stress-associated intestinal inflammation. As such, they represent promising candidate for maintaining intestinal barrier function.

A common challenge in the production of hydrolysates is the development of undesirable bitterness during enzymatic hydrolysis, which adversely affects their sensory quality and consumer acceptance [16]. Microbial fermentation can significantly improve the bitter taste of rice bran protein hydrolysates. Lactic acid bacteria (LAB) are a group of microorganisms that produce lactic acid as the primary end product of carbohydrate fermentation [17]. They include the genera *Lactobacillus*, *Streptococcus*, and *Lactococcus*. The lactic acid generated via LAB during fermentation contributes to improved sensory characteristics, including taste and flavor, of rice bran protein hydrolysates [18]. In addition, LAB can produce many metabolites during fermentation, and these metabolites also possess a variety of biological activities that improve fermentation products activities [19]. Thus, co-treatment with enzymes and LAB represents a promising strategy for simultaneously improving the sensory qualities and biological activity of rice bran protein hydrolysates.

This study employed enzymatic-LAB co-treatment technology to prepare rice bran protein hydrolysates and subsequently characterized the structural features of the hydrolysates produced via Alcalase hydrolysis (RHP) and those generated via synergistic treatment with Alcalase and *L. plantarum* 13110 (CRB). This study was also designed to evaluate the protective potential of rice bran protein hydrolysates in a mouse model with DSS-induced ulcerative colitis, thereby establishing a theoretical foundation for their use in mitigating intestinal mucosal damage.

## 2. Materials and Methods

### 2.1. Materials and Reagents

Rice bran protein powder (protein content 85%) and Alcalase (24,000 U/mL) were obtained from Shaanxi Panier Biotechnology Co., Ltd. (Xi’an, China) and Novozymes Biotechnology Co., Ltd. (Bagsvaerd, Denmark), respectively. *Lactiplantibacillus plantarum* 13110 (CGMCC No. 24693) was deposited in the China Common Microbial species preservation Center (Beijing, China).

### 2.2. Preparation of RHP and CRB

After preparing a 13% (*w*/*v*) rice bran protein solution and adjusting its pH to 8.5, it was hydrolyzed at 60 °C for three hours with Alcalase at a concentration of 700 U/g. Following the reaction, the solution was boiled in water for ten minutes, cooled to room temperature, and then centrifuged at 4000× *g* for fifteen minutes. The rice bran protein (RHP) hydrolysate was obtained by freeze-drying the supernatant and storing it.

After hydrolysis, the reaction mixture was sterilized at 121 °C for 20 min using a vertical pressure steam sterilizer (SX-500, TOMY Digital Biology Co., Ltd., Tokyo, Japan). *Lactiplantibacillus plantarum* 13110 was then inoculated at an optimized concentration, and the pH was adjusted to the desired value. Fermentation was carried out at a controlled temperature for a specified duration to obtain the fermentation broth. To determine the optimal fermentation conditions for CRB preparation, a two-stage experimental design was employed: single-factor-at-a-time (OFAT) experiments followed by an orthogonal experiment design. Specifically, each parameter was optimized sequentially while keeping the others constant at their baseline levels. The following ranges were tested: initial pH (4.5, 5.0, 5.5, 6.0, and 6.5), inoculum size (0%, 1%, 2%, 3%, 4%, and 5%, *v*/*v*), fermentation temperature (31, 34, 37, 40, and 43 °C), and fermentation time (12, 24, 36, 48, and 60 h). Following fermentation, the mixture was centrifuged at 8000× *g* for 15 min, and the supernatant was collected and freeze-dried to obtain the co-treated rice bran (CRB) protein hydrolysate.

Based on the single-factor results, three levels for each of the three factors were selected for the subsequent orthogonal experiment. An L_9_(3^4^) orthogonal array was adopted to identify the optimal combination of fermentation conditions and to evaluate the relative importance of each factor, including pH (5.0, 5.5, and 6.0), inoculum size (1%, 2%, and 3%, *v*/*v*), and fermentation temperature (31, 34, and 37 °C). After each optimization step, the newly determined optimal value was used in subsequent experiments. The final optimal conditions were identified based on the highest DPPH and ABTS radical scavenging activities. Prior to analysis, the CRB was filtered through a 0.22 μm microporous membrane.

### 2.3. Fourier Transform Infrared Spectrum of RHP and CRB

Sampling was performed according to the potassium bromide pressing tablet method described by Hadidi et al. [20]. Specifically, a 1 mg sample was weighed, and KBr powder was added. The mixture was then mixed ground and compressed into a solid film, and the resulting film was scanned 32 times in the 4000–400 cm^−1^ band at a 4 cm^−1^ resolution.

### 2.4. UV Spectrum Analysis of RHP and CRB

Following the method described in a previous study [21], UV absorption spectra were recorded using a UV-Vis spectrophotometer (Lambda 650, PerkinElmer Co., Ltd., Springfield, IL, USA) over a wavelength range of 200–600 nm. The scanning parameters were set as follows: a scan rate of 100 nm/min, a bandwidth of 5.0 nm, a data interval of 1.0 nm, a response time of 0.2 s, and a path length of 1 cm. All measurements were performed at 25 °C, and each sample was scanned in triplicate to obtain the final UV absorption spectrum.

### 2.5. Molecular Weight Distributions of RHP and CRB

The molecular weight distributions of RHP and CRB were determined via gel filtration chromatography using a Superdex Peptide 10/300 GL column (GlycoScience Life Technology Co., Ltd., Shanghai, China). Blue Dextran 2000 was employed to determine the void volume (V_0_), and the column was calibrated using the following molecular weight standards: reduced glutathione (307 Da), oxidized glutathione (612 Da), bacitracin (1422 Da), and aprotinin (6511 Da). Standard proteins with known molecular weights were used to generate a calibration curve, in which the effective partition coefficient (K_a_w) served as the abscissa, while the logarithm of molecular weight (log Mr) served as the ordinate. Based on this curve, the relative molecular masses of RHP and CRB were calculated.

### 2.6. Zeta Potential Determination of RHP and CRB

RHP and CRB samples were prepared as 1 mg/mL solutions in deionized water, and their zeta potentials were determined using a Malvern Nano-ZS90 laser particle size analyzer (Malvern Instruments Limited, Malvern, UK). Measurements were conducted at 25 °C over a period of 120 s, with 20 repeated readings performed using a DTS1070 cell.

### 2.7. Amino Acid Composition of RHP and CRB

For amino acid analysis, approximately 0.06 g of the sample (based on protein content) was weighed into an ampoule tube and hydrolyzed with 9 mL of 6.67 mol/L HCl. The tube was purged with nitrogen for 10 min, sealed, and heated at 110 °C for 24 h. After cooling, the hydrolysate was adjusted to pH 2.2 with 8.5 mL of 6 mol/L NaOH, filtered, and subjected to analysis using an automatic amino acid analyzer (L-8900, Hitachi Co., Ltd., Tokyo, Japan).

### 2.8. Determination of DPPH Radical Scavenging Activity

The DPPH radical scavenging activity was assessed using the method described by Peng et al. [22].

### 2.9. Determination of ABTS Free Radical Scavenging Activity

The ABTS radical scavenging activity of RHP and CRB (0.5 mg/mL) was determined according to [23].

### 2.10. Experimental Animals and Design

**Figure 1:**
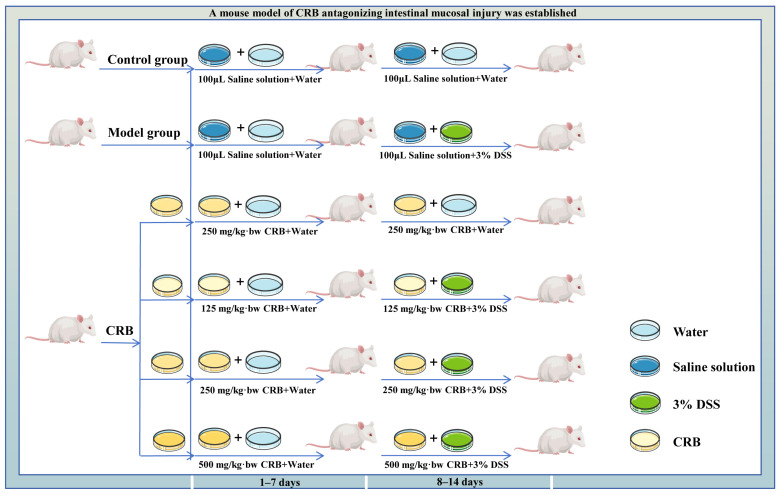
Schematic illustration of the animal experimental design and treatment protocol. Male Kunming mice (*n* = 60) were randomly divided into six groups (*n* = 10 per group) after a 7–day acclimatization period. During the first 7 days (days 1–7), all mice received normal drinking water and daily intragastric administration of either normal saline or different doses of CRB, as indicated. From day 8 to day 14, colitis was induced by replacing drinking water with 3% (*w*/*v*) DSS in all groups except the Control group and the CRB medium-dose (without DSS) group, which continued to receive normal water. Throughout the 14–day experimental period, body weight and fecal characteristics were monitored daily. On day 14, after the final gavage and a 12–h fast, all mice were euthanized for sample collection. DSS: dextran sulfate sodium; CRB: co-treated rice bran protein hydrolysate (Alcalase and *L. plantarum* 13110).

Sixty healthy male Kunming mice aged 6–8 weeks were selected, and after 7 days of acclimatization, they were randomly assigned to six groups (*n* = 10 per group): control, DSS model, medium-dose CRB without DSS, low-dose CRB (125 mg/kg·bw), medium-dose CRB (250 mg/kg·bw), and high-dose CRB (500 mg/kg·bw). The randomization sequence was generated by a researcher who was not involved in subsequent animal handling or data collection. Random allocation was performed at the individual animal level. All animals were housed in standard polypropylene cages under identical environmental conditions (12 h light/dark cycle, 22 ± 2 °C, 50–60% humidity) with ad libitum access to standard chow and drinking water (or DSS solution). The experimental timeline is illustrated in Figure 1. The experiment was conducted in a single-blinded manner. The investigator responsible for daily gavage administration was aware of the group allocation to ensure correct dosing. All mice received a normal diet throughout the study period. Colitis was induced through ad libitum administration of 3% (*w*/*v*) dextran sulfate sodium (DSS) in drinking water from day 8 to day 14, except for the control group and the medium-dose CRB (without DSS) group, which received sterile water throughout. CRB was administered daily via gavage at the indicated doses for 14 consecutive days, while the control and DSS model groups received an equal volume of saline. Body weight and fecal characteristics were monitored daily. On day 14, after the final gavage, mice were fasted for 12 h prior to blood collection from the orbital sinus, and serum was subsequently separated. The animals were then euthanized via cervical dislocation, and colon tissues were harvested and stored at −80 °C for subsequent analyses. Data analysis was also performed by a statistician who was unaware of group assignments.

### 2.11. Disease Activity Index (DAI) Score

DAI scores were expressed as scores for weight change, stool viscosity, and hematochezia. The specific criteria are listed in Table 1.

### 2.12. Histopathological Observation

For histopathological evaluation, colon specimens were fixed in 10% neutral buffered formalin for 24 h, then processed for paraffin embedding, sectioning, and H&E staining. The stained sections were examined microscopically to evaluate key parameters, including inflammatory cell infiltration, lesion extent, crypt structure, and epithelial damage.

### 2.13. Spleen Index Determination

Spleens were excised from the mice, rinsed with normal saline, blotted dry, and weighed. The spleen index was calculated according to Formula (1).

(1) $$\mathrm{Spleen}\;\mathrm{index}\;\left(\%\right)=\frac{\mathrm{Weight}\;\mathrm{of}\;\mathrm{spleen}\;(\mathrm{mg})}{\mathrm{Body}\;\mathrm{weight}\;\left(g\right)}\;\times \;100$$

### 2.14. Determination of Biochemical Indexes of Colon Tissue and Serum

To prepare tissue homogenates, 100 mg of colon tissue was homogenized in ice-cold water to achieve a 10% (*w*/*v*) concentration. After centrifugation at 4000 rpm for 15 min at 4 °C, the supernatant was recovered and transferred to a clean EP tube. The indicators were measured using commercial ELISA kits (Jianglai Biotechnology Co., Ltd., Shanghai, China) according to the manufacturer’s instructions, including superoxide dismutase activity (SOD), glutathione peroxidase activity (GSH-Px), malondialdehyde content (MDA), tumor necrosis factor-α content (TNF-α), interleukin-6 content (IL-6), interleukin-1β content (IL-1β), interleukin-10 content (IL-10), lipopolysaccharides content (LPSs), fatty acid-binding protein 2 content (FABP2), D-lactic acid content (D-LA), diamine oxidase activity (DAO), Myeloperoxidase activity (MPO).

### 2.15. Statistical Analysis

All data were analyzed using IBM SPSS Statistics 26 software and expressed as the mean ± standard deviation (SD). Statistical comparisons were performed using one-way analysis of variance (ANOVA), followed by Tukey’s honestly significant difference (HSD) post hoc test for pairwise comparisons. All figures were generated using Origin 2019 software. For animal experiments, results are presented as the mean ± SD (*n* = 10 per group), and different lowercase letters indicate significant differences among groups (*p* < 0.05). Effect sizes for pairwise comparisons were calculated as Cohen’s d. Values of 0.2, 0.5, and 0.8 were considered small, medium, and large effects, respectively.

## 3. Results and Discussion

### 3.1. Synergistic Treatment with Alcalase and Lactiplantibacillus plantarum 13110 Significantly Enhanced the Antioxidant Activity

The synergistic action of protease and lactic acid bacteria (LAB) facilitates further degradation of macromolecular rice bran proteins, yielding a greater abundance of small-molecule active peptides that are more readily absorbed [24]. Notably, different LAB strains produce distinct metabolites, leading to variations in the physiological activities of the peptides generated during fermentation, which in turn affects the antioxidant activity of the final products [25]. In our previous study, we compared the antioxidant activities of rice bran protein hydrolysates prepared using four different commercial proteases (Alcalase, Papain, Neutrase, and Flavourzyme). Among these, Alcalase-derived hydrolysates exhibited the highest DPPH and ABTS radical scavenging activities. Alcalase is a serine protease from *Bacillus licheniformis* that preferentially cleaves peptide bonds at the carboxyl side of hydrophobic amino acid residues (e.g., Phe, Trp, Tyr, Leu, Val), generating peptides with potent antioxidant properties. Based on these preliminary results, Alcalase was selected as the primary enzyme for the initial hydrolysis step in this study, and further determined the optimal hydrolysis condition (RHP) for Alcalase, so it was fermented using LAB. Specifically, *L. plantarum* 13110, *L. plantarum* 09002, *L. plantarum* 02002, *L. paracasei*, and *L. bulgaricus* were used to ferment RHP. The antioxidant activity of the co-fermentation products prepared using *L. plantarum* 13110 was significantly improved compared with the RHP (*p* < 0.05), as the bacterium may produce abundant aminopeptidase and carboxypeptidase that act on RHP, potentially causing more peptides with ABTS and DPPH free radical scavenging activities to be released. In addition, LAB also produce certain bioactive substances during fermentation, such as extracellular polysaccharides, whose synergistic effects enhance the antioxidant activity [2]. Due to these factors, the ABTS and DPPH free radical scavenging rates of *L. plantarum* 13110’s collaborative products are significantly higher than those of other products obtained via LAB fermentation. Compared with Alcalase hydrolysates, the bitterness from the fermentation process was greatly reduced, and the flavor was significantly improved. Therefore, we selected *L. plantarum* 13110 as the best strain for co-fermenting of enzyme-LAB.

To obtain hydrolysates with enhanced antioxidant activity, the fermentation conditions of *L. plantarum* 13110 on the hydrolysates were optimized using antioxidant activities as evaluation indicators. Based on the range analysis of the L_9_(3^4^) orthogonal experiment, the optimal fermentation conditions were determined as follows: an inoculum size of 2% (*v*/*v*, 10^8^ CFU/mL), a fermentation temperature of 31 °C, an initial pH of 5.5, and a fermentation time of 36 h. Under these conditions, the ABTS radical scavenging rate reached 90.35 ± 0.1% (at 0.5 mg/mL), the DPPH radical scavenging rate reached 57.42 ± 2.0% (at 1 mg/mL), and the protein recovery rate was 53.51 ± 0.8%, meaning that approximately 46.5% of the initial rice bran protein was not recovered in the final freeze-dried supernatant. *L. plantarum* 13110 is a metabolically active microorganism that requires a carbon and nitrogen source for growth. During the 36-h fermentation period, the bacteria may utilize part of the soluble peptides and free amino acids as a carbon source in addition to a nitrogen source, especially when the available carbohydrates in the rice bran are limited. This insoluble pellet, which was discarded in the present study, could potentially be valorized in future applications. For example, it may be used as a protein-rich supplement in animal feed. Alternatively, it could be re-solubilized under alkaline conditions and subjected to further enzymatic treatment to improve overall yield.

The antioxidant activity of the CRB was significantly higher than that of previously reported plant-derived protein hydrolysates, including walnut protein active peptides [26], rice protein hydrolysates (RPHs) [27], and wheat seed protein antioxidant peptides [28]. At the same protein concentration, CRB exhibited 14.08% and 21.30% increases in ABTS and DPPH radical scavenging rates, respectively, compared with hydrolysates prepared using Alcalase alone. The improved antioxidant activity of CRB was closely associated with *L. plantarum* 13110 fermentation, which synergistically degraded RHP into lower-molecular-weight hydrolysates—a finding subsequently confirmed via molecular weight distribution analysis.

### 3.2. Fourier Transform Infrared Spectroscopy

Fourier transform infrared (FTIR) spectroscopy, which combines the Fourier transform with infrared spectroscopy, enables the identification of functional groups and the determination of secondary structures in proteins [29]. The characteristic absorption bands associated with protein secondary structures include the amide I band (1700–1600 cm^−1^), arising primarily from C=O stretching vibrations coupled with C–N stretching along the peptide backbone; the amide II band (1600–1500 cm^−1^), attributed to C–N stretching and N–H bending vibrations; and the amide III band (1330–1220 cm^−1^), corresponding to C–N stretching and N–H in-plane bending vibrations [29].

**Figure 2:**
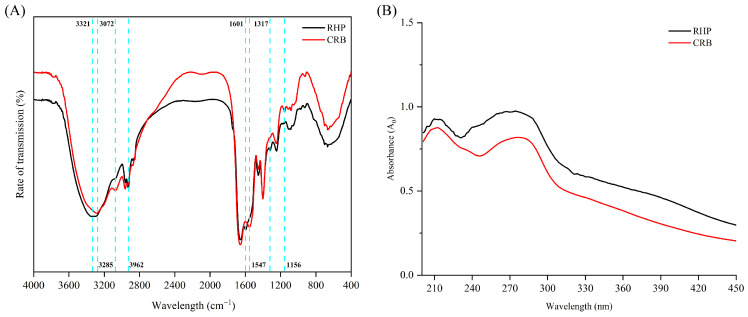
Structural characterization of RHP and CRB. (**A**) FTIR spectra (4000–400 cm^−1^) showing characteristic amide I, II, and III bands. Fermentation-induced structural changes are reflected in altered peak intensities and positions. (**B**) UV spectra (190–450 nm) of RHP and CRB. RHP: rice bran protein hydrolysate (Alcalase hydrolysis alone); CRB: co-treated rice bran protein hydrolysate (Alcalase and *L. plantarum* 13110).

The FTIR spectra of RHP and CRB are significantly different. RHP displays N–H bond stretching vibration absorption peaks at 3321 cm^−1^, 3072 cm^−1^, and 2962 cm^−1^, which belong to the amide A and amide B bands (Figure 2A). Compared with RHP, the intensity of the three absorption peaks of CRB changes significantly, and the absorption peak at 3321 cm^−1^ shows a red shift, which may be due to the combination of free O–H tensile vibration and hydrogen bonds in the CRB structure after *L. plantarum* 13110 fermentation [30]. The amide I band is mainly generated through C=O vibration in the amide group and is sensitive to changes in the secondary structure, so it is of great significance in the evaluation of protein secondary structures [28]. The absorption peak of CRB changed at 1601 cm^−1^, resulting in a red shift. The change in this interval was attributed to the amide I band (1700–1600 cm^−1^), which was mainly caused by C=O stretching and C–N stretching vibration, indicating that CRB’s secondary structure changed. This interval change in the amide III band occurs at 1317 cm^−1^, which mainly results from the change in N–H bending vibration and C–N stretching vibration. Compared with RHP, CRB changed at 1156 cm^−1^, and a new absorption peak was generated, which may be caused by the interaction between the newly generated metabolites and the amide group in RHP [31]. These results showed that after fermenting of RHP with *L. plantarum* 13110, the protein chain was further disrupted and new molecules were generated, which had positive effects on CRB’s antioxidant activity.

### 3.3. UV Spectrum Results

The characteristic UV absorption of proteins arises primarily from the side chains of aromatic amino acid residues, specifically tryptophan, tyrosine, and phenylalanine, which absorb light in the ultraviolet region [32]. As shown in Figure 2B, comparative analysis revealed two characteristic absorption peaks for both samples: one at 225 nm, primarily attributable to peptide bond absorption, and another at 280 nm, corresponding to π→π* transitions in the aromatic side chains of phenylalanine residues [33]. A decrease in UV absorption intensity was observed, suggesting structural alterations in CRB following fermentation. These UV spectral findings further corroborate that the fermentation process induced conformational changes in the hydrolysates.

### 3.4. Molecular Weight Distribution (MWD)

The antioxidant activity of bioactive peptides is closely correlated with their molecular weight, with peptides of different molecular masses exhibiting significant differences in antioxidative capacity. In the present study, the MWD of RHP and CRB were determined using gel filtration chromatography to characterize and compare the peptide profiles of the two hydrolysates [34].

**Figure 3:**
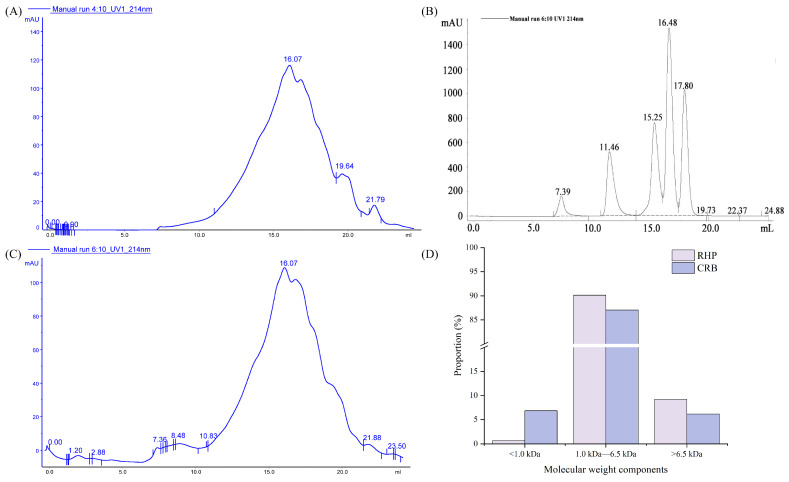
Molecular weight distribution elution of RPH and CRB. (**A**,**B**) Gel filtration chromatograms of RHP and CRB monitored at 214 nm. (**C**) Elution profile of molecular weight standards: aprotinin (6511 Da), bacitracin (1422 Da), oxidized glutathione (612 Da), and reduced glutathione (307 Da). (**D**) Quantitative comparison of fractions in three molecular weight ranges (<1.0 kDa, 1.0–6.5 kDa, and >6.5 kDa). Chromatography was performed on a Superdex Peptide 10/300 GL column. RHP: rice bran protein hydrolysate (Alcalase hydrolysis alone); CRB: co-treated rice bran protein hydrolysate (Alcalase and *L. plantarum* 13110).

Their molecular weight distributions are presented in Figure 3. The RHP consisted of 0.67% (<1.0 kDa), 90.11% (1.0–6.5 kDa), and 9.22% (>6.5 kDa) fractions. In comparison, the CRB contained 6.84%, 87.02%, and 6.14% of components in the corresponding molecular weight ranges. It can be seen that after RHP fermentation, the proportion of macromolecular components decreased, while the proportion of small molecular components increased significantly. Compared with RHP, the content of fractions <1.0 kDa in CRB was significantly increased (*p* < 0.05). In our previous study, we found that *L. plantarum* 13110 had high protease activity in fermentation broth and could specifically degrade rice bran protein. During fermentation, the protease secreted by *L. plantarum* 13110 can further degrade the substrate and reduce its molecular weight, and fermentation with *L. plantarum* 13110 resulted in a higher abundance of small molecular weight peptides exhibiting strong antioxidant activity [35]. Alcalase, a serine endoprotease from *Bacillus licheniformis*, cleaves internal peptide bonds primarily at the carboxyl side of hydrophobic residues (e.g., Phe, Trp, Tyr, Leu, Val, Ile). This initial endoproteolytic attack generates a mixture of medium-sized peptides, which constitute the majority of RHP. However, Alcalase alone has limited ability to further degrade these medium-sized peptides into smaller fragments because it lacks significant exopeptidase activity. In contrast, *L. plantarum* 13110 produces a range of aminopeptidases and carboxypeptidases, which sequentially remove amino acids from the *N*-terminus and *C*-terminus of peptides, respectively. These exopeptidases can act on the peptides generated by Alcalase, progressively trimming them into smaller fragments. Importantly, the endoproteolytic products of Alcalase expose new *N*- and *C*-termini that serve as preferred substrates for bacterial exopeptidases, creating a synergistic degradation cascade. The increase of the <1.0 kDa fraction in CRB compared with RHP directly reflects this combined endo-/exopeptidase action. It explains why CRB exhibited significantly higher antioxidant activity than RHP, as smaller peptides are generally more readily absorbed and often possess greater radical-scavenging capacity.

### 3.5. Zeta Potential Analysis

For proteins, the zeta potential is directly influenced by the net charge of amino acid residues exposed on the molecular surface [36]. Specifically, a predominance of positively charged residues results in a positive zeta potential, while an abundance of negatively charged residues gives rise to a negative zeta potential. The zeta potential values of RHP versus CRB are shown in Table 2, and their values were all negative, indicating that the protein surface contained a large amount of negative charge. CRB’s zeta potential value decreased from 43.31 mV to 19.22 mV, which may be due to the change in the hydrolysates component’s structure during the fermentation process, as well as exposure of the internal positively charged group, thereby neutralizing the negative charge in the solution, weakening the electrostatic interaction, and increasing the stability of the CRB component.

### 3.6. Amino Acid Composition Analysis

**Figure 4:**
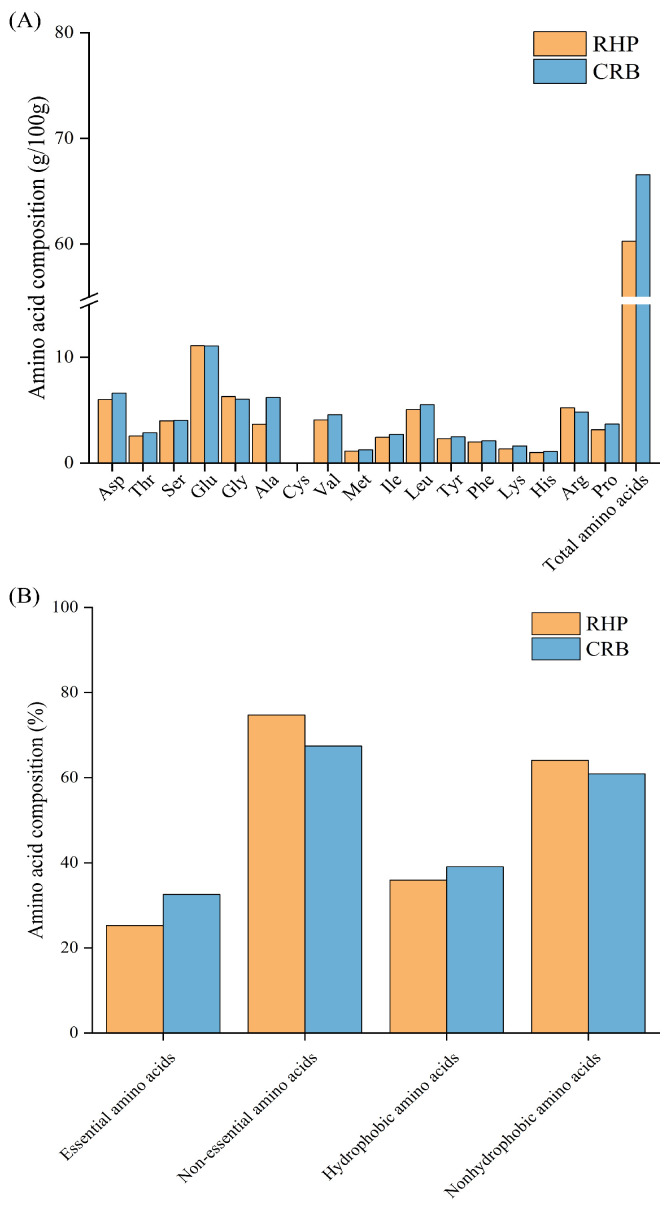
Amino acid composition of RHP and CRB. (**A**) seventeen Amino acid profiles were determined by automatic amino acid analysis after acid hydrolysis. Data are expressed as mg/g protein. (**B**) Quantitative comparison of essential amino acids, non-essential amino acids, hydrophobic amino acids, and nonhydrophobic amino acids between RHP and CRB. RHP: rice bran protein hydrolysate (Alcalase hydrolysis alone); CRB: co-treated rice bran protein hydrolysate (Alcalase and *L. plantarum* 13110).

Sixteen different amino acids were detected in RHP and CRB. Among them, the EAA/TAA and EAA/NEAA ratios of RHP and CRB were in line with the reference protein pattern recommended by the FAO/WHO [37], indicating that the nutritional value of rice bran protein was retained. As shown in Figure 4, the total amino acid content of CRB was 66.57 g/100 g higher than that of RHP (60.28 g/100 g), and the proportion of hydrophobic amino acids was 39.07% higher than that of RHP (35.95%). During the fermentation process of *L. plantarum* 13110, the substrate’s insoluble macromolecular proteins were further decomposed into soluble hydrolysates, which improved the utilization of rice bran protein-derived raw material. Compared with RHP, protein recovery from CRB was improved, which in turn resulted in greater changes in its amino acid composition. Specifically, hydrolysates containing elevated levels of Lys, Leu, Val, Glu, and Ala have been widely reported to exhibit strong antioxidative effects [38]. The amount of Ala, Val, Lys, and Leu in the amino acid composition of CRB was higher than that of RHP, which may be one of the reasons why the antioxidant capacity of CRB was significantly better than that of RHP. Therefore, in the subsequent study, we selected CRB with better in vitro antioxidant activity for animal experiments to explore the antagonistic mechanism of CRB on ulcerative colitis mice.

### 3.7. Antagonistic Mechanisms of CRB on Ulcerative Colitis

#### 3.7.1. CRB Attenuates Body Weight Loss and Reduces DAI Score in Colitic Mice

**Figure 5:**
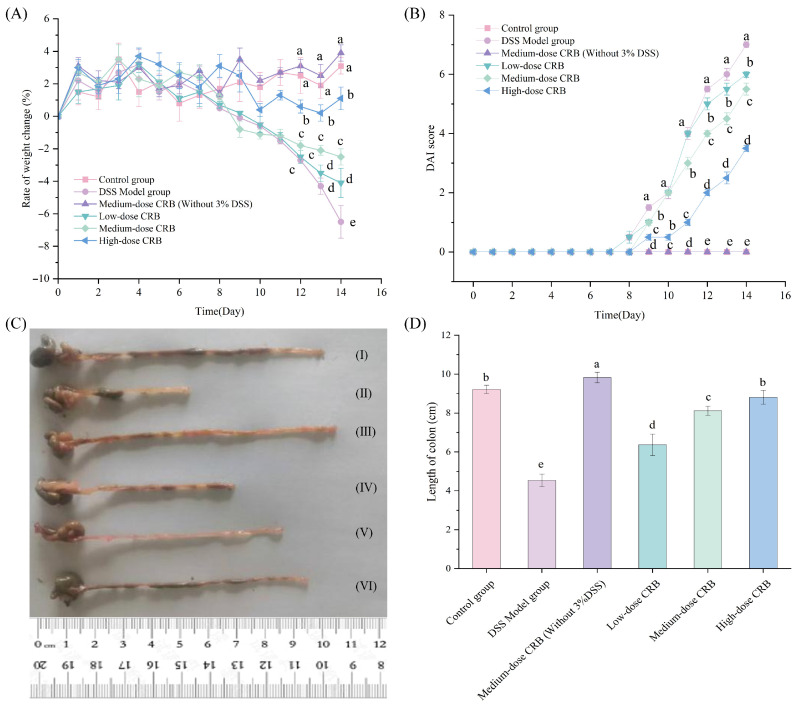
Effects of CRB on body weight change, DAI score, and colon length in DSS-induced colitic mice. (**A**) Body weight change (%) over 14 days. (**B**) Disease activity index (DAI) score. (**C**) Representative colon images from each group at day 14: (I) Control, (II) DSS model, (III) medium-dose CRB without DSS, (IV) low-dose CRB, (V) medium-dose CRB, (VI) high-dose CRB. (**D**) Colon length measured at sacrifice. Data are presented as the mean ± SD (*n* = 10). Different lowercase letters indicate significant differences among groups on the same day (**A**,**B**) (*p* < 0.05). Different lowercase letters indicate significant differences between groups (**D**) (*p* < 0.05). DSS: dextran sulfate sodium; CRB: co-treated rice bran protein hydrolysate (Alcalase and *L. plantarum* 13110).

Body weight variations and DAI scores serve as important parameters for assessing the clinical severity of ulcerative colitis [39], so body weight and stool consistency were monitored daily for all groups, and scores were assigned based on the criteria outlined in Table 1. As can be seen from Figure 5A,B, during the first 1–7 days of the experiment, all mice had a small rate of weight change, a normal diet, drinking water, and activity status, and a DAI score of 0. From the 8th day, the weight of mice in the DSS model group began to decrease, especially from the 12th day to the 14th day, during which a straight downward trend in the weight change rate was observed. This finding was significantly different from that of the control group (*p* < 0.05). However, in terms of DAI score, the activity of the DSS model mice decreased, and the hair color was rough, dim, listless, and unresponsive. The DAI score showed an upward trend, and there was a significant difference compared with the control group. In the late stage of DSS model establishment, the mice gradually appeared to have loose stools, mucoid stools, and bloody stools, which were also typical symptoms of ulcerative colitis in mice. The above phenomena indicated that the mouse DSS colitis model was successfully established. Treatment with low-, medium-, and high-dose CRB significantly attenuated body weight loss and reduced DAI scores compared with the DSS model group (*p* < 0.05, d = 0.98, 0.97, 0.92). These effects were accompanied by improved diarrhea status, reduced hematochezia, and enhanced overall conditions, particularly in the high-dose group. All CRB-treated groups showed significant differences in body weight change and DAI scores relative to the DSS model group (*p* < 0.05), indicating that CRB effectively alleviated clinical symptoms of colitis.

#### 3.7.2. CRB Treatment Attenuated DSS-Induced Colon Shortening in a Dose-Dependent Manner

The colonic length of the ulcerative colitis mice was significantly shortened. Their intestinal wall was congested, and their stool was thick, shapeless, bloody, and even loose [40]. Their colonic inflammation status could be determined based on the colon length.

As shown in Figure 5C,D, compared with the control group, the colon length of the DSS model group was significantly shortened (*p* < 0.05, d = 0.99), from 9.22 ± 0.21 cm to 4.54 ± 0.32 cm, which further indicated that the DSS model mice were successfully established. CRB intervention dose-dependently restored colon length in DSS-induced colitic mice relative to those in the DSS model group. These findings demonstrate that CRB effectively inhibits intestinal stress responses and improves colon length in mice with ulcerative colitis, which may be due to the higher antioxidant activity of CRB in vitro. In addition, CRB administration alone (medium dose without 3% DSS) did not induce any significant alterations relative to the control group, further supporting its safety profile. Similar observations were reported by Chen et al. [41], who found that millet gliadin peptides exerted protective effects against ulcerative colitis in mice without adverse effects.

#### 3.7.3. Effect of CRB on Histopathology in Mice with Ulcerative Colitis

**Figure 6:**
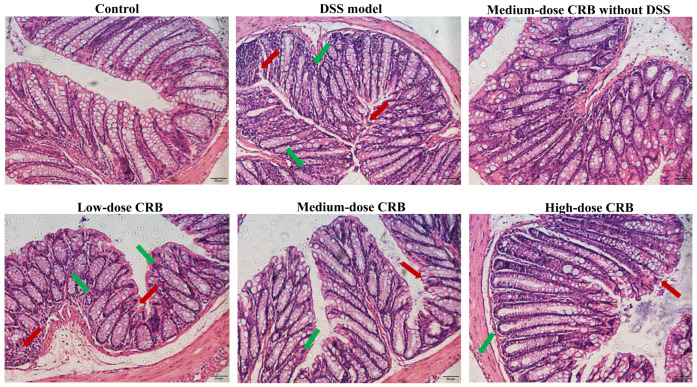
Effects of CRB on colonic pathology in mice (200×, scale bar = 50 μm). The red arrow indicates the crypt structure, and the green arrow indicates the inflammatory cells. DSS: dextran sulfate sodium; CRB: co-treated rice bran protein hydrolysate (Alcalase and *L. plantarum* 13110).

The colon tissue sections of mice were prepared via HE staining to observe the mice’s colon tissue conditions. As shown in Figure 6, the colonic tissue structure of the control group and the CRB medium-dose group (without 3% DSS) was complete, the intestinal glands of the mucosal layer were abundant and arranged regularly and evenly, the submucosal structure was close, and no pathological damage occurred. Mice in the DSS model group showed marked colonic damage, including mucosal edema, glandular necrosis, and inflammatory cell infiltration, confirming the successful induction of colitis by DSS. Compared with the DSS model group, the morphology of colon tissue cells in all CRB groups was significantly improved. Although an improvement in colonic tissue structure and cell morphology was observed in the low-dose CRB group, loosely arranged cells and residual necrotic epithelial cells were still evident. The most pronounced amelioration of colonic histoarchitecture was observed in the high-dose CRB group. This group displayed near-normal colonic architecture with intact structure, regular cell morphology, and minimal damage, comparable to the control group. This confirms that CRB effectively mitigates DSS-induced colonic damage and confers significant protection against colitis. Future studies should include blinded evaluation by a qualified gastrointestinal pathologist using validated, semi-quantitative scoring instruments to ensure the highest level of objectivity and reproducibility.

#### 3.7.4. Effect of CRB on the Spleen Index in Mice with Ulcerative Colitis

The spleen index is an important index to evaluate immune damage in experimental animals. When the body has an inflammatory response, the spleen, as an immune organ, will be enlarged, and the spleen index will increase significantly [42]. Therefore, the degree of inflammatory injury can be evaluated by measuring the spleen index of mice.

**Figure 7:**
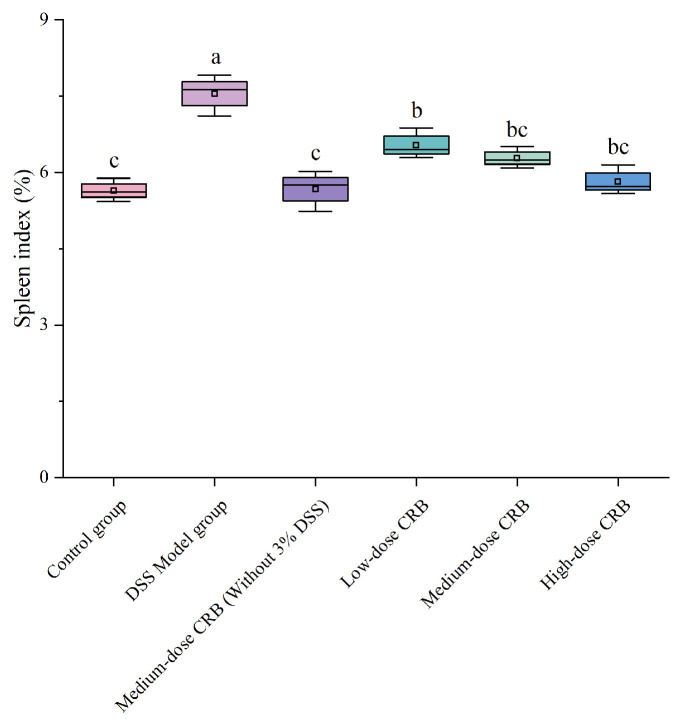
Effects of CRB on the spleen index, data are presented as the mean ± SD (*n* = 10). Different lowercase letters represent significant differences between groups (*p* < 0.05, one-way ANOVA followed by Tukey’s HSD post hoc test). For each boxplot, the box represents the interquartile range (25th–75th percentiles), the horizontal line indicates the median, and whiskers extend to the minimum and maximum values. DSS: dextran sulfate sodium; CRB: co-treated rice bran protein hydrolysate (Alcalase and *L. plantarum* 13110).

Compared with the control group, the DSS model group showed a significantly increased spleen index (*p* < 0.05, d = 0.94), reflecting severe splenomegaly (Figure 7). Administering CRB at medium- and high- doses significantly reversed this effect, reducing the spleen index by 16.82% and 22.91%, respectively, relative to the DSS model group (*p* < 0.05, d = 0.88, 0.92). These results demonstrate that CRB effectively mitigates splenomegaly and suppresses the inflammatory response in the colon, thereby exerting protective effects against DSS-induced colitis.

#### 3.7.5. Effects of CRB on Oxidative Stress in Mice with Ulcerative Colitis

When DSS is induced in mice, it can stimulate colon cells, induce excessive colon cell-derived oxygen free radical production, aggravate cellular lipid peroxidation, destroy cell membrane structure, lead to blood flow disorder of the colon mucosa, and cause intestinal mucosa damage. Compared with the control group, the DSS model group exhibited significantly decreased GSH-Px (285.48 U/g, d = 0.86) and SOD activities (140.38 U/g, d = 0.98) (*p* < 0.05), accompanied by a significantly increase in MDA content (20.64 nmol/g, d = 0.85) (*p* < 0.05). Oxidative stress was successfully established in mouse colon tissue following DSS administration, as demonstrated by these findings.

**Figure 8:**
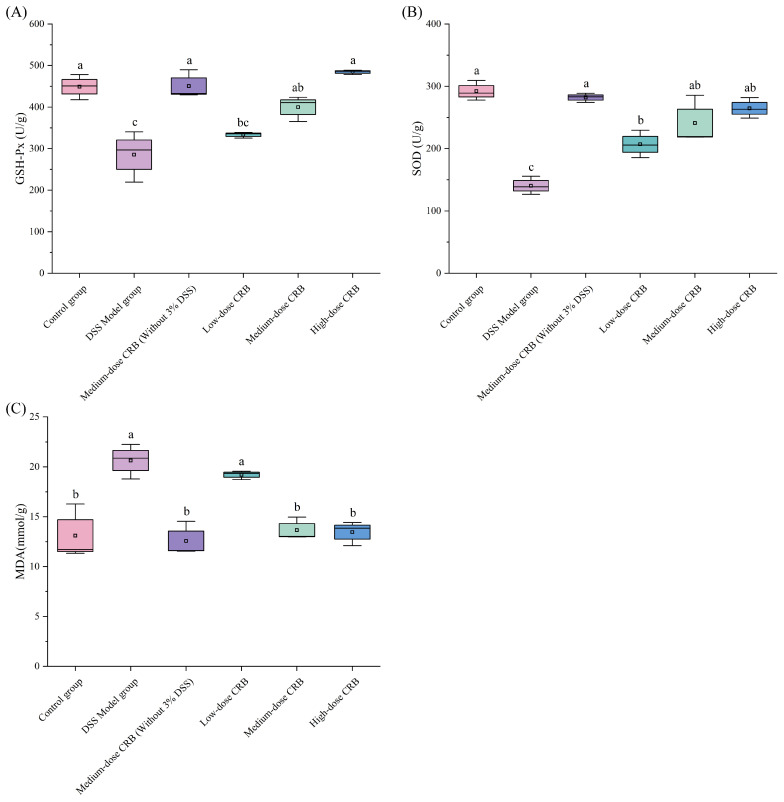
Effects of CRB on oxidative stress in mice with ulcerative colitis. (**A**) the activity of GSH-Px, (**B**) the activity of SOD, and (**C**) the content of MDA. Data are presented as the mean ± SD (*n* = 10). Different lowercase letters represent significant differences between groups (*p* < 0.05, one-way ANOVA followed by Tukey’s HSD post hoc test). For each boxplot, the box represents the interquartile range (25th–75th percentiles), the horizontal line indicates the median, and whiskers extend to the minimum and maximum values. DSS: dextran sulfate sodium; CRB: co-treated rice bran protein hydrolysate (Alcalase and *L. plantarum* 13110).

Glutathione peroxidase (GSH-Px), an important peroxidolytic enzyme widely present in the body, can scavenge oxygen free radicals and resist lipid peroxidation of intestinal mucosal epithelial cells, thereby protecting tissues from damage [43]. According to Figure 8A, following DSS induction, GSH-Px activity in the model group was significantly decreased to 285.48 U/g compared with the control group (*p* < 0.05, d = 0.86), indicating that DSS treatment depleted GSH-Px enzyme activity and compromised the antioxidant capacity of colon tissue. CRB treatment dose-dependently elevated colonic GSH-Px activity relative to the DSS model group, with increases of 28.60%, and 41.08% in the medium-, and high-dose groups, respectively (*p* < 0.05, d = 0.76, 0.91). Furthermore, GSH-Px levels in all CRB-treated groups were restored to levels comparable with the control group.

As a naturally occurring antioxidant enzyme, superoxide dismutase (SOD) is ubiquitously expressed in animals, plants, and microorganisms. It plays a key role in cellular defense by catalyzing the dismutation of superoxide radicals into oxygen and hydrogen peroxide [44]. Studies have found that SOD can convert cell-derived superoxide free radicals into hydrogen peroxide, thereby reducing the oxidative stress level in them and protecting cells them [45]. Compared with the DSS model group (Figure 8B), each CRB dose group could increase SOD activity in colon tissue. The administration of high-dose CRB significantly increased colonic SOD activity by 46.96% compared with the DSS model group (*p* < 0.05, d = 0.97), with no significant difference from the control group (*p* > 0.05).

As a major product of lipid peroxidation, malondialdehyde (MDA) not only reflects oxidative damage but also perpetuates membrane injury by oxidizing plasma membrane components, thereby compromising tissue and cellular structures [44]. Rezaie et al. reported that MDA content in tissues reflects the extent of membrane structural damage, serving as a reliable biomarker of lipid peroxidation and oxidative injury [46]. CRB treatment at medium- and high- doses significantly decreased colonic MDA content by 33.87% and 34.79%, respectively, relative to the DSS model group (*p* < 0.05, d = 0.92, 0.92), indicating attenuation of lipid peroxidation (Figure 8C). MDA levels in the low-dose CRB group remained similar to those in the DSS model group. In contrast, high-dose CRB administration normalized the MDA content, with values indistinguishable from the control group (*p* > 0.05, d = 0.08). These results demonstrate that medium- and high-dose CRB effectively scavenged excessive free radicals generated in colon tissue, restored colonic antioxidant capacity, and alleviated lipid peroxidation on colonocyte membranes. This protective effect may be attributed to the potent free radical scavenging activity of CRB.

In summary, CRB exerts its protective effects against colonic damage through two mechanisms: enhancing endogenous antioxidant enzyme activities (GSH-Px and SOD) to bolster systemic antioxidant capacity, and inhibiting lipid peroxidation on colon cell membranes to attenuate oxidative stress. These actions collectively contribute to the amelioration of colonic mucosal injury. Consistent with our findings, Jing et al. [47] demonstrated that corn protein hydrolysates (500 mg/kg·bw) significantly enhanced colonic GSH-Px and SOD activities in DSS-induced colitic mice, thereby alleviating oxidative stress in the UC model.

#### 3.7.6. Effects of CRB on the Inflammatory Response of Colonic Tissue in Mice

**Figure 9:**
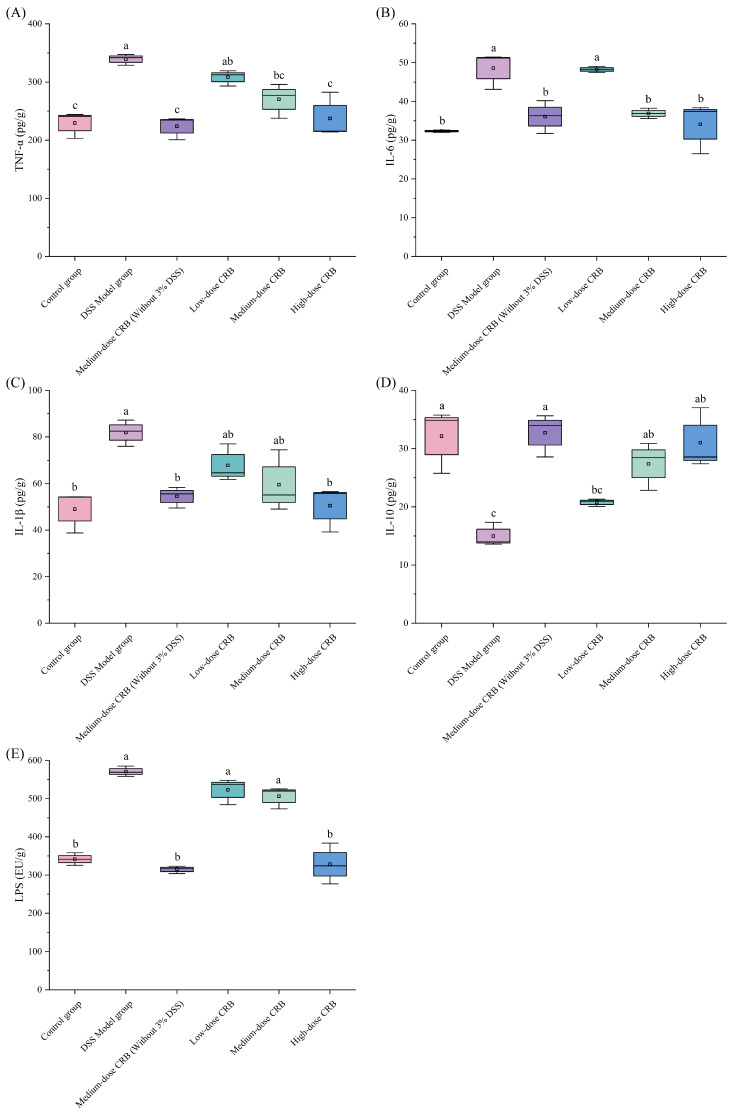
Effects of CRB on the inflammatory response of colonic tissue in mice. (**A**) The expression levels of TNF-α, (**B**) IL-6, (**C**) IL-1β, (**D**) IL-10, and (**E**) LPSs. Data are presented as the mean ± SD (*n* = 10). Different lowercase letters represent significant differences between groups (*p* < 0.05, one-way ANOVA followed by Tukey’s HSD post hoc test). For each boxplot, the box represents the interquartile range (25th–75th percentiles), the horizontal line indicates the median, and whiskers extend to the minimum and maximum values. DSS: dextran sulfate sodium; CRB: co-treated rice bran protein hydrolysate (Alcalase and *L. plantarum* 13110).

DSS induction can cause an inflammatory response in colon tissue, directly damage colonic mucosal cells, and destroy the colonic mucosal barrier. It can also activate the body’s immune system and stimulate the production of cellular inflammatory factors, such as TNF-α and IL-1β. Figure 9 illustrates that DSS treatment significantly upregulated colonic levels of TNF-α (339.36 pg/g, d = 0.99), IL-6 (48.59 pg/g, d = 0.92), IL-1β (81.91 pg/g, d = 0.91), and LPS (571.02 EU/g, d = 0.99), while downregulating IL-10 (14.98 pg/g, d = 0.91) compared with the control group. These results showed that CRB intervention could effectively alleviate the inflammatory level in mice.

Tumor necrosis factor-α (TNF-α) is a proinflammatory factor widely involved in the body’s inflammatory response. Additionally, anti-tumor necrosis factor-α monoclonal antibodies have been used to treat inflammatory bowel disease for many years, as they have the advantages of rapidly inducing and maintaining disease remission, reducing complications, and achieving mucosal healing [48]. As shown in Figure 9A, DSS induction significantly elevated TNF-α release in the colon tissue of the model group compared with the control group. However, intervention with different doses of CRB effectively reduced colonic TNF-α levels relative to the DSS model group. The most pronounced effect was observed in the high-dose CRB group, in which TNF-α release was decreased by 30.04%, reaching a level comparable to that of the control group with no statistically significant difference (*p* < 0.05, d = 0.87). These findings demonstrate that CRB intervention effectively suppresses TNF-α production and alleviates the inflammatory response in colon tissue.

As a multifunctional cytokine, interleukin-6 (IL-6) regulates cell growth and differentiation and contributes to anti-infective immune responses. Clinically, IL-6 serves as a reliable inflammatory biomarker, as its expression levels mirror parallel the degree of inflammation [49]. Compared with the DSS model group (Figure 9B), different doses of CRB in the experimental groups had alleviating effects on IL-6 production in colon tissue. Intervention with medium- and high-dose CRB significantly reduced IL-6 levels in colon tissue compared with the DSS model group (*p* < 0.05, d = 0.86, 0.78). Specifically, the IL-6 content in the high-dose CRB group decreased by 29.88%. These findings indicate that CRB effectively suppresses IL-6 release in colon tissue, thereby contributing to the attenuation of the inflammatory response.

As a major mediator of inflammation, interleukin-1β (IL-1β) is mainly derived from monocytes and macrophages. Additionally, under antigenic stimulation, neutrophils, plasma cells, and other nucleated cells can contribute to its synthesis and release [50]. Daniel et al. found that IL-1β expression was significantly increased in diseased colonic tissues of ulcerative colitis patients [51]. As shown in Figure 9C, the high-dose CRB group had the best effect, as CRB intervention significantly decreased colonic IL-1β levels by 29.89% relative to the DSS model group (*p* < 0.05, d = 0.89), with no significant difference from the control group (*p* > 0.05, d = 0.07). This demonstrates that CRB effectively reduces pro-inflammatory cytokine (IL-6 and IL-1β) release, mitigating neutrophil infiltration and colonic inflammation.

As a key immunoregulatory cytokine, interleukin-10 (IL-10) plays a pivotal role in suppressing inflammation by inhibiting the production and secretion of pro-inflammatory cytokines from macrophages, thereby counteracting inflammatory progression [52]. Figure 9D shows that compared with the DSS model group, different doses of CRB intervention could effectively promote the release of the anti-inflammatory factor IL-10 in colon tissues. The high-dose group had the best effect, and the release of IL-10 in colon tissue increased by106.94% (*p* < 0.05, d = 0.89). These results indicate that CRB intervention can effectively increase the IL-10 content in colon tissue and inhibit the release of macrophage-derived inflammatory factors, thereby reducing the inflammatory response in colon tissue.

Lipopolysaccharides (LPSs), a kind of endotoxin, are the main component of the Gram-negative bacterial cell wall. Anand et al. [53] found that LPSs can destroy the tight junction between cells, leading to an increase in the permeability of the intestinal mucosal barrier, which in turn promotes bacterial and endotoxin invasion and causes intestinal inflammation. Figure 9E illustrates that DSS induction significantly increased colonic LPS levels to 571.02 EU/g relative to the control group (*p* < 0.05, d = 0.99). The LPS content in the high-dose group was comparable to that of the control group, demonstrating that CRB potently inhibits colonic LPS accumulation and thus alleviates inflammatory responses. The effect of CRB on DSS-induced colonic mucosal barrier injury is particularly obvious in the high-dose groups, which further indicates that CRB plays a certain role in inflammatory response attenuation (*p* < 0.05, d = 0.95). In addition, feeding with CRB alone did not show any significant changes in any of the parameters compared with the control group.

The present study demonstrates that CRB not only exhibits potent antioxidant activity in vitro but also exerts significant anti-inflammatory effects in vivo, as evidenced by its protective role in a DSS-induced colitis mouse model. These two effects are not independent and are linked by a potential biological association. Oxidative stress and inflammatory responses form a self-amplifying vicious cycle in the pathological progression of ulcerative colitis. Reactive oxygen species and other oxidizing agents can directly activate pro-inflammatory signaling pathways and induce cellular damage. Conversely, persistent inflammatory responses recruit and activate additional immune cells, leading to further escalation of oxidative stress levels and exacerbating tissue injury. Therefore, the anti-inflammatory effects observed in vivo in this study are likely attributable in part to CRB’s ability to rapidly and effectively block the initiation phase of this cycle through its direct antioxidant activity, such as scavenging free radicals and enhancing the activity of endogenous antioxidant enzymes. In other words, the antioxidant function of CRB may serve as the primary mechanism and crucial foundation for its anti-inflammatory effects within the body. Although oxidative stress and inflammatory pathways are highly intertwined biologically and antioxidant interventions often result in anti-inflammatory effects, this study provides insufficient evidence to conclude that its anti-inflammatory action is entirely or primarily mediated by its direct antioxidant capacity. Therefore, the findings of this study more accurately reveal a functional association. CRB possesses dual biological activities, namely antioxidant and anti-inflammatory properties. To clarify its underlying mechanism of action, future studies should employ additional mechanistic experiments. For instance, researchers should validate whether it retains anti-inflammatory effects when free radical scavenging is inhibited in cellular models, or utilize transcriptomic/proteomic analyses to identify its core regulatory pathway networks. In summary, the current findings provide strong support for developing CRB as a functional dietary ingredient with both antioxidant and anti-inflammatory potential. However, its precise mechanisms of action and the causal pathways are yet to be further elucidated.

#### 3.7.7. Effects of CRB on FABP2 and D-LA Contents and DAO Level in Serum

DSS induction can cause damage to the colonic mucosal barrier in mice, inhibit the expression of fatty acid-binding protein 2 (FABP2), reduce the expression level of FABP2 in serum, and increase the colon permeability in mice. DAO was released by a large number of necrotic colonic mucosal epithelial cells, and D-lactic acid content (D-LA) produced by intestinal flora metabolism entered the blood through the intercellular space of the intestinal wall. The expression levels of D-LA and DAO in serum were increased. To evaluate CRB’s the protective effect against colonic mucosal injury in mice, FABP2 and D-LA contents and DAO activity in serum were measured.

**Figure 10:**
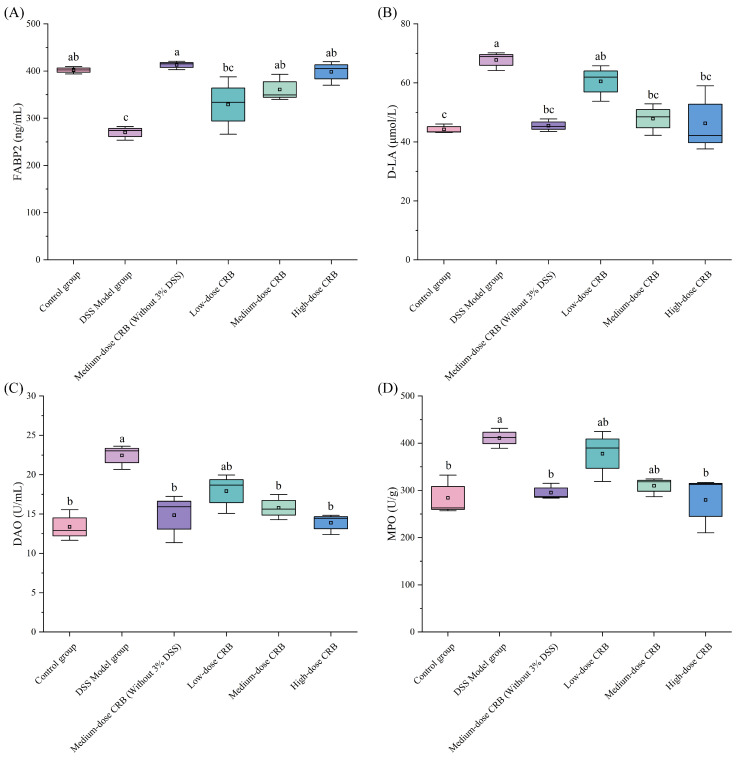
Effects of CRB on (**A**) serum FABP2 content, (**B**) serum D-LA content, (**C**) serum DAO activity, and (**D**) MPO activity in colonic tissue. Data are presented as the mean ± SD (*n* = 10). Different lowercase letters represent significant differences between groups (*p* < 0.05, one-way ANOVA followed by Tukey’s HSD post hoc test). For each boxplot, the box represents the interquartile range (25th–75th percentiles), the horizontal line indicates the median, and whiskers extend to the minimum and maximum values. DSS: dextran sulfate sodium; CRB: co-treated rice bran protein hydrolysate (Alcalase and *L. plantarum* 13110).

Figure 10 shows that DSS induction significantly altered the serum levels of FABP2, D-LA, and DAO compared with the control group, reflecting DSS-induced colonic inflammation and barrier disruption. CRB treatment at medium and high doses markedly increased FABP2 content while decreasing D-LA levels and DAO activity relative to the DSS model group). Specifically, in the high-dose group, FABP2 increased from 270.02 to 399.21 ng/mL (d = 0.98), D-LA decreased from 67.77 to 46.25 μmol/L (d = 0.97), and DAO activity dropped from 22.44 to 13.89 U/mL (d = 0.93).

FABP2 is an important member of the fatty acid binding protein superfamily, which is mainly involved in the absorption, transport, redistribution, and utilization of fatty acids in organelles. Research has revealed a strong correlation between FABP2 and the pathogenesis of inflammatory diseases as well as intestinal ischemic injury [54]. As can be seen from Figure 10A, the FABP2 content in the serum of the model group was significantly reduced in mice with DSS-induced intestinal mucosal injury (*p* < 0.05), indicating impaired FABP2 synthesis in inflamed enterocytes. CRB treatment at medium and high doses significantly increased serum FABP2 content relative to the DSS model group (*p* < 0.05, d = 0.89, 0.95). The most pronounced effect was observed in the high-dose group, in which serum FABP2 levels were restored to near-normal levels, representing an increase of 47.84% compared to the model group, suggesting recovery of enterocyte functional integrity. Although some studies have reported elevated serum FABP2 levels in DSS-induced colitis models [54], the present study found a significant decrease in serum FABP2 in the model group. Under physiological conditions, mature intestinal epithelial cells continuously synthesize FABP2, maintaining a basal serum level. DSS challenge may reduce FABP2 synthesis through two synergistic mechanisms, directing suppression of *Fabp2* gene transcription via inhibition of peroxisome proliferator activated receptor γ (PPARγ)-dependent activity, and induction of rapid epithelial turnover, which increases the proportion of immature cells that express low levels of FABP2. Together, these processes lead to a severely impaired capacity for FABP2 production and a consequent decrease in its serum concentration. Following CRB intervention, PPARγ activity is restored, and epithelial differentiation and barrier function are gradually repaired, allowing FABP2 synthesis to return to normal levels. This dynamic process highlights the dual nature of FABP2 as a functional marker rather than merely a passive indicator of epithelial damage, and more importantly, suggests that the restoration of serum FABP2 reflects a global recovery of intestinal epithelial cell function.

D-LA is the final product of intestinal bacterial metabolism, but there is no enzyme system that can rapidly decompose D-lactic acid in mammals. Studies have shown that the increase in D-lactic acid level is related to the extent of intestinal mucosal barrier disfunction [55]. As shown in Figure 10B, CRB intervention at medium and high doses significantly decreased serum D-LA content compared with the DSS model group (*p* < 0.05, d = 0.91, 0.79). The high-dose group exhibited the most pronounced effect, with a 31.75% reduction (*p* < 0.05, d = 0.79), and D-LA levels were not significantly compared with the control group. These results indicated that CRB can effectively inhibit colonic inflammation, reduce the permeability of the colonic mucosal barrier, and reduce serum D-LA content.

As an intracellular enzyme, diamine oxidase (DAO) is mainly distributed in the mucosa and ciliated epithelial cells of the mammalian small intestine, where it plays an important role in catalyzing the breakdown of diamines. It can promote cell repair and protect the intestinal mucosa. Under normal circumstances, DAO expression in the intestinal tissue is very low, but when the intestinal mucosa is damaged, it will increase abnormally [56]. CRB intervention at medium- and high- doses significantly decreased DAO activity compared with the DSS model group (*p* < 0.05, d = 0.90, 0.94), with no significant difference from the control group (Figure 10C). These findings demonstrate that CRB effectively reduces oxidative stress in colon tissue, protects membrane integrity, and restores DAO release to physiological levels.

The intestinal barrier damage markers D-LA and DAO enable quantitative assessment of CRB’s protective efficacy against barrier dysfunction [57]. The results showed that CRB intervention in the ulcerative colitis mouse model significantly reduces the content of D-LA and DAO, which play protective roles in the intestinal barrier. In conclusion, CRB could promote colonic mucosal barrier repair by promoting FABP2 expression in mice. On the other hand, it can regulate the intestinal immune response and inhibit the inflammatory response by improving the intestinal flora environment in mice. Furthermore, it can reduce the D-LA and DAO levels in mice and alleviate DSS-induced colonic mucosal barrier injury.

#### 3.7.8. Effect of CRB on MPO Activity in Mouse Colonic Tissue

Myeloperoxidase (MPO) is a heme-containing enzyme belonging to the heme peroxidase superfamily. It is primarily secreted by neutrophils, monocytes, and tissue-resident macrophages, and it is expressed in high concentrations in neutrophils. External stimulation can cause neutrophils to aggregate, release MPO, and cause tissue cell damage in inflammatory sites. Studies have found that the level of MPO activity can reflect the number and degree of neutrophil infiltration in a certain tissue and indirectly reflect the degree of inflammation in the tissue [58]. O’Neill et al. [59] found that MPO expression is often increased in the inflammatory mucosa of ulcerative colitis patients, which can promote cancer, as it is related to the degree of ulcerative colitis inflammation to a certain extent. Therefore, MPO activity can be used to determine the severity of colitis inflammation.

As shown in Figure 10D, compared with the control group, MPO activity in the DSS model group was significantly increased to 410.98 U/g (*p* < 0.05, d = 0.88), which indicated that a large number of neutrophils were infiltrated and activated in the colon tissue of DSS-induced mice. Compared with the DSS model group, MPO activity in the high-dose CRB groups decreased significantly by 31.88% (*p* < 0.05, d = 0.82), respectively, and there was no significant difference compared with the control group. These results suggested that CRB could alleviate DSS-induced colonic mucosal barrier injury and inflammatory responses by reducing neutrophils infiltrations in mouse colon tissues, thereby reducing MPO release. This further indicates that reducing MPO levels, which reduces damage to the colonic mucosal barrier, is one of the ways that CRB intervention alleviates DSS-induced colonic mucosal injury.

This study systematically investigated the protective effects of rice bran protein hydrolysate (CRB) produced synergistically using Alcalase and *L. plantarum* 13110 against DSS-induced ulcerative colitis. Existing research confirms that plant protein hydrolysates alleviate colitis symptoms by downregulating pro-inflammatory factors such as TNF-α, IL-1β, and IL-6, upregulating anti-inflammatory factors like IL-10, and repairing intestinal barrier function [60,61]. This mechanism aligns with CRB’s pathway of regulating inflammatory factor balance and reducing intestinal barrier damage markers such as D-LA and DAO. However, unlike strategies relying solely on enzymatic hydrolysis to prepare proteolytic hydrolysates, this study innovatively employs a synergistic enzymatic hydrolysis probiotic fermentation process. This approach significantly enhances the antioxidant activity of CRB compared to products derived using enzymatic hydrolysis alone (RHP). Regarding target mechanisms, CRB reduces oxidative damage by enhancing SOD and GSH-Px activity while simultaneously targeting intestinal barrier function markers such as FABP2, D-LA, and DAO. This approach distinctly differs from existing studies that evaluate tight junction proteins as primary indicators [62]. Furthermore, compared to the phenomenon where ovomucoid hydrolysate exhibits varying “beneficial/harmful” effects due to different enzymatic hydrolysis methods [63], CRB’s synergistic processing strategy consistently demonstrates a protective effect, further validating the critical impact of process optimization on the functionality of hydrolysates. Although our current findings indicate that CRB possesses efficacy in alleviating colitis, its molecular mechanisms require further elucidation. Moving forward, we will screen CRB for active peptide segments exhibiting significant anti-inflammatory effects. We will then conduct further investigations using methods such as qRT-PCR, Western blotting, and immunohistochemical staining to dissect the signaling pathways involved.

The present study confirms that rice bran protein hydrolysates possess significant antioxidant and anti-inflammatory activities in vivo, providing a scientific basis for its high-value utilization in the food industry. This hydrolysate has potential as a natural functional ingredient for the development of gut-health-promoting foods, such as beverages and yogurt. Furthermore, it offers new insights for the formulation of nutritional support products targeting inflammatory bowel disease in the field of foods for special medical purposes. Collectively, the conversion of rice bran protein hydrolysate into value-added raw materials not only aligns with resource sustainability strategies but also opens innovative avenues for the development of functional foods that support intestinal health.

## 4. Conclusions

Collectively, our results show that *L. plantarum* 13110 fermentation enhances the antioxidant properties of rice bran protein hydrolysates, and that CRB exerts protective effects against DSS-induced colitis by mitigating oxidative stress and inflammation. This study provides a scientific rationale for utilizing rice bran protein in anti-colitis strategies. Further studies are warranted to identify the CRB-derived bioactive peptide sequences that are responsible for its anti-colitis effects and to elucidate the molecular mechanisms through which CRB enhances intestinal barrier function.

## Figures and Tables

**Table 1:** Standard of DAI scores.

Score	Weight Loss (%)	Consistency of Stool	Hematochezia
0	0	Normal	Normal
1	1–5	Soft stools	Minor bleeding
2	6–10	Slightly loose stools	There was perianal blood
3	11–15	Mucoid stool	Gross bloody stool
4	>16	Loose fluid stool	Heavy bleeding

**Table 2:** RHP’s and CRB’s zeta potentials.

Sample	Zeta Potential (mV)
RHP	−43.31 ± 0.19 a
CRB	−19.22 ± 0.27 b

Note: All experiments were performed in triplicate independently. Data are presented as the mean ± standard deviation (SD) (*n* = 3). Different lowercase letters indicate statistically significant differences between groups (*p* < 0.05). RHP: rice bran protein hydrolysate (Alcalase hydrolysis alone); CRB: co-treated rice bran protein hydrolysate (Alcalase and *L. plantarum* 13110).

## Data Availability

The original contributions presented in this study are included in the article. Further inquiries can be directed to the corresponding authors.
